# Proinflammatory cytokine responses induced by influenza A (H5N1) viruses in primary human alveolar and bronchial epithelial cells

**DOI:** 10.1186/1465-9921-6-135

**Published:** 2005-11-11

**Authors:** MCW Chan, CY Cheung, WH Chui, SW Tsao, JM Nicholls, YO Chan, RWY Chan, HT Long, LLM Poon, Y Guan, JSM Peiris

**Affiliations:** 1 Department of Microbiology, The University of Hong Kong, Queen Mary Hospital, Hong Kong Special Administrative Region of China; 2 Department of Cardiothoracic Surgery, Grantham Hospital, Wong Chuk Hang, Aberdeen, Hong Kong Special Administrative Region of China; 3 Department of Anatomy, The University of Hong Kong, Pokfulam, Hong Kong Special Administrative Region of China; 4 Department of Pathology, The University of Hong Kong, Queen Mary Hospital, Hong Kong Special Administrative Region of China; 5 National Institute of Hygiene and Epidemiology, Hanoi, Vietnam

**Keywords:** avian, chemokines, IP-10, pathogenesis

## Abstract

**Background:**

Fatal human respiratory disease associated with influenza A subtype H5N1 has been documented in Hong Kong, and more recently in Vietnam, Thailand and Cambodia. We previously demonstrated that patients with H5N1 disease had unusually high serum levels of IP-10 (interferon-gamma-inducible protein-10). Furthermore, when compared with human influenza virus subtype H1N1, the H5N1 viruses in 1997 (A/Hong Kong/483/97) (H5N1/97) were more potent inducers of pro-inflammatory cytokines (e.g. tumor necrosis factor-a) and chemokines (e.g. IP-10) from primary human macrophages *in vitro*, which suggests that cytokines dysregulation may play a role in pathogenesis of H5N1 disease. Since respiratory epithelial cells are the primary target cell for replication of influenza viruses, it is pertinent to investigate the cytokine induction profile of H5N1 viruses in these cells.

**Methods:**

We used quantitative RT-PCR and ELISA to compare the profile of cytokine and chemokine gene expression induced by H5N1 viruses A/HK/483/97 (H5N1/97), A/Vietnam/1194/04 and A/Vietnam/3046/04 (both H5N1/04) with that of human H1N1 virus in human primary alveolar and bronchial epithelial cells *in vitro*.

**Results:**

We demonstrated that in comparison to human H1N1 viruses, H5N1/97 and H5N1/04 viruses were more potent inducers of IP-10, interferon beta, RANTES (regulated on activation, normal T cell expressed and secreted) and interleukin 6 (IL-6) in primary human alveolar and bronchial epithelial cells *in vitro*. Recent H5N1 viruses from Vietnam (H5N1/04) appeared to be even more potent at inducing IP-10 than H5N1/97 virus.

**Conclusion:**

The H5N1/97 and H5N1/04 subtype influenza A viruses are more potent inducers of proinflammatory cytokines and chemokines in primary human respiratory epithelial cells than subtype H1N1 virus. We suggest that this hyper-induction of cytokines may be relevant to the pathogenesis of human H5N1 disease.

## Background

Influenza pandemics arise from genetic reassortment between avian and human influenza viruses or alternatively by the direct adaptation of a avian influenza viruses to efficient human-to-human transmission [1]. Avian influenza A subtype H5N1 transmitted from poultry to humans in Hong Kong in 1997 (H5N1/97) causing fatal human respiratory disease [2,3]. The subsequent re-emergence of human H5N1 disease in southern China [4], Vietnam [5], Thailand and Cambodia [6] has raised the specter of a new influenza pandemic. While human-to-human transmission of the H5N1 subtype influenza virus appears to be inefficient so far, the disease has exceptional severity in those affected with reported mortality rates ranging from 33% in Hong Kong in 1997 to 55% in Thailand and Vietnam in 2004. The reasons for this unusual severity of human disease have remained unclear.

While dissemination outside the respiratory tract was not demonstrated in human H5N1 disease in 1997 and 2003 [4,7], there is some evidence that more recent H5N1 viruses may occasionally disseminate to multiple organs contributing to unusual disease manifestations such as meningo-encephalitis [8]. However, most patients with H5N1 disease had a primary viral pneumonia complicated by the syndromes of acute respiratory distress and multiple organ dysfunction [4-7,9] with lymphopenia and haemophagocytosis being notable findings. The syndromes of acute respiratory distress and multiple organ dysfunction as well as haemophagocytosis have previously been associated with cytokine dysregulation [10,11].

Influenza virus infection of blood-monocyte-derived murine and human [12,13] macrophages and porcine alveolar macrophages [14] have been shown to result in induction of pro-inflammatory cytokines. Furthermore, we have previously demonstrated that, when compared to human H1N1 and H3N2 influenza viruses, infection of H5N1/97-like viruses lead to the hyper-induction of proinflammatory cytokines in human primary macrophage cultures *in vitro *[12]. We also reported that patients with H5N1 disease have unusually high serum concentrations of chemokines IP-10 (interferon-gamma-inducible protein-10) and MIG (monokine induced by interferon γ) [4]. We have therefore hypothesized that this differential hyper-induction of cytokines and chemokines may contribute to the unusual severity of human H5N1 disease [4,12].

While macrophages are a key sentinel cell of the immune system and are permissive to influenza virus replication, the primary target cell for the virus are respiratory epithelial cells [15]. In primates experimentally infected with H5N1/97 virus, the type I and II pneumocytes and alveolar macrophages were found to contain viral antigen [16]. Virus infection of alveolar pneumocytes was also demonstrated in the lung of a patient with fatal H5N1 disease [17]. Human alveolar epithelial cells are vital for the maintenance of lung function and the pulmonary air-blood barrier. In addition, human respiratory epithelial cells respond to viral infections by mounting a cytokine response that contributes both to the innate and adaptive host defenses [18]. Furthermore, type II pneumocytes express class II major histocompatibility complex (MHC) molecules *in vivo *[19]. Expression of class II MHC is usually limited to specialized cells of the immune system whose role is to present foreign antigen to helper T cells [20,21]. The expression of these molecules on alveolar epithelial cells is likely to be of relevance to the adaptive immune response. Therefore it is important to study cytokine responses induced by infection of epithelial cells with influenza viruses including H5N1 viruses.

Human influenza A viruses have been previously reported to induce interleukin 6 (IL-6), interleukin 8 (IL-8) and RANTES (regulated on activation, normal T cell expressed and secreted) *in vitro *from the transformed bronchial epithelial cell line (NCI-H292) [18]. However, the physiological relevance of findings from transformed cell lines is uncertain and primary alveolar epithelial cell cultures would be a more relevant model [22]. Here, we have compared the cytokine profiles induced by H5N1/97 and H1N1 viruses in human primary type II pneumocytes and bronchial epithelial cells *in vitro *to test the hypothesis that H5N1/97 and H5N1/04 viruses differentially hyper-induce pro-inflammatory cytokines in respiratory epithelial cells.

## Materials and methods

### Viruses

An influenza virus isolated from a patient with fatal influenza A H5N1 disease in Hong Kong in 1997, A/Hong Kong/483/97 (H5N1/97), viruses from patients with H5N1 disease in Vietnam in 2004, A/Vietnam/1194/04 and A/Vietnam/3046/04 (both abbreviated as H5N1/04) and a human H1N1 virus A/Hong Kong/54/98 (H1N1) were studied. Viruses were initially isolated in Madin-Darby canine kidney (MDCK) cells. They were cloned by limiting dilution, and seed virus stocks were prepared in MDCK cells. Virus infectivity was assessed by titration of tissue culture infection dose 50% (TCID_50_) in MDCK cells. The H5N1 influenza viruses used in this study were handled in a BL3 biocontainment facility.

### Cells

Primary human bronchial epithelial cells (NHBE) were obtained from Cambrex Bio Science (Walkersville, Inc., Maryland, USA). NHBE cells were grown according to the suppliers instructions in serum-free and hormone supplemented bronchial epithelial growth media (BEGM) which included supplements of 13 g/l bovine pituitary extract, 0.5 g/l hydrocortisone, 0.5 mg/l human recombinant epidermal growth factor, 0.5 g/l epinephrine, 10 g/l transferrin, 5 g/l insulin, 0.1 mg/l retinoic acid, 6.5 mg/l 3,3',5-triiodo-L-thryonine, 50 g/l gentamicin, and 50 mg/l amphotericin B (Cambrex Bio Science, Walkersville, Inc., Maryland, USA). Medium was changed daily starting from the day after seeding. Cells reached confluency in approximately 9 to 10 days, and nearly confluent cells were subcultured using trypsin/EDTA (Cambrex) at a ratio of 1:5. Experiments were carried out on the same batch of cells at passage 3 to 4. The cells were incubated in a humidified atmosphere (5% CO_2_, 37°C) under liquid-covered conditions.

Primary human alveolar epithelial cells (type II pneumocytes) were isolated from human non-tumor lung tissue obtained from 13 patients (mean age 65 yr [range, 46–77 yr], 10 males and 3 females) undergoing lung resection in Grantham Hospital, Hong Kong. The research protocol was approved by the ethics committee of the University of Hong Kong and Hospital Authority Hong Kong West Cluster. Human type II pneumocytes were isolated using a modification of the methods previously described [19,23]. Briefly, after removing visible bronchi, the lung tissue was chopped into pieces of >0.5 mm thickness using a tissue chopper, washed with balanced salt solution (BSS, 137 mM NaCl, 5 mM KCl, 0.7 mM Na_2_HPO_4_, 10 mM HEPES, 5.5 mM glucose, pH 7.4) for 30 min at 37°C three times to partially remove macrophages and blood cells. The tissue was digested using a combination of trypsin (0.5%, GIBCO BRL, Gaithersburg, MD, USA) and elastase (2 units/ml, Worthington Biochemical Corporation, Lakewood, NJ, USA) twice for 15 min at 37°C in a shaking water-bath. The partially digested tissue was minced in the presence of 40% fetal bovine serum (FBS) in DMEM/F12 medium and DNase I (350 units/ml) (GIBCO BRL, Gaithersburg, MD, USA), and cell clumps dispersed by repeatedly pipetting the cell suspension for 10 minutes. After filtration through gauze and a 40 μm cell strainer to ensure a single cell suspension, the cells were incubated with a 1:1 mixture of DMEM/F12 medium and small airway growth medium (SAGM, Cambrex Bio Science Walkersville, Inc., Maryland, USA) containing 5% FBS and 350 units/ml DNase I, on tissue-culture treated plastic Petri dishes in a humidified incubator (5% CO_2_, 37°C) for 2 hours in order to let macrophage attach on the plastic surface. The non-adherent cells were layered on a discontinuous Percoll density gradient (densities 1.089 and 1.040 g/ml) and centrifuged at 25 × g for 20 min. The cell layer at the interface of the two gradients was collected and washed four times with BSS to remove the Percoll. To remove remaining alveolar macrophages, the cell suspension was incubated with magnetic beads coated with anti-CD-14 antibodies at room temperature for 20 min under constant mixing. After the removal of the beads using a magnet and assessment of cell viability by trypan-blue exclusion, the purified type II pneumocyte suspension was suspended in SAGM supplemented with 1% FBS, 100 units/ml penicillin and 100 μg/ml streptomycin, and plated at a cell density of 300,000 cells/cm^2^. The cells were maintained in a humidified atmosphere (5% CO_2_, 37°C) under liquid-covered conditions, and growth medium was changed daily starting from 60 hours after plating the cells.

### Characterization of human type II pneumocytes

#### Staining for alkaline phosphatase

Human type II pneumocytes were identified by staining for alkaline phosphatase. Freshly isolated cells were spun down on glass slides, air-dried, and stained for 20 min at room temperature. The stain was prepared by dissolving 10 mg naphthol AS bi-phosphate (Sigma) in 40 μl DMSO and was diluted in 10 ml of 0.125 M 2-amino-2-methyl propanol buffer (pH 8.9, Sigma) containing 10 mg fast red (Sigma). The slide was washed and counterstained in 1% methylene green (Sigma) for 30 seconds and was mounted in aqueous medium [19].

#### Transmission electron microscopy

Cells were fixed in 2% glutaraldehyde (Electron Microscopy Sciences, Washington, PA, USA), washed three times in phosphate buffered saline and serially dehydrated in acetone. The tissue was post-fixed in 1% osmium tetroxide and embedded in an Araldite resin (Polysciences, Inc., Washington, PS, USA). Semi-thin sections (1 μm) were cut using an ultra-microtome (Reichert Ultracut S, Leica Aktiengesellscharft, Wien, Australia) with a diamond knife and were stained with toluidine blue for light microscopic examination. Ultra-thin sections (80 nm) mounted on copper grids were electron contrasted with uranyl acetate (1.5 hours, 30°C, Electron Microscopy Sciences) and lead citrate (40 minutes, 20°C, Electron Microscopy Sciences, Washington, PA, USA), and were examined with a transmission electron microscope (EM 208S, FEI Company, Hillsboro, Oregon, USA).

#### Flow cytometry

The expression of cell surface antigen was measured by staining purified type II pneumocytes with optimal dilution of rabbit anti-human surfactant protein-C (SP-C) (Upstate, Lake Placid, NY, USA) monoclonal antibodies (24°C, 30 minutes) followed by a fluorescein isothiocyanate (FITC-conjugated goat anti-mouse IgG antibody; Sigma, F-0257, 24°C, 30 minutes). Each cell preparation was also stained with antibody specific for monocyte/macrophage surface antigen (CD14 conjugated with FITC, MCA2185F; Serotec. Oxford, UK). The cells were examined by the flow cytometry (FACSSCalibur; Becton Dickinson), and the FITC-stained cells were detected by measuring green light emitted at 530 nm (FL1 channel). The percentage of cells expressing the epithelial and macrophage makers were determined.

### Influenza virus infection of type II pneumocytes and bronchial epithelial cells

Human type II pneumocytes and bronchial epithelial cells (seeded at 1 × 10^6 ^cells per well in 24-well tissue-culture plates) were infected at a multiplicity of infection (MOI) of two unless otherwise indicated. After 60 min of virus adsorption, the virus inoculum was removed, and the cells were washed with warm culture medium (SAGM for type II pneumocytes and BEBM for bronchial epithelial cells) and incubated in medium supplemented with 0.6 mg/L penicillin, 60 mg/L streptomycin, and 2 mg/L N-p-tosyl-L-phenylalanine chloromethyl ketone-treated-trypsin (Sigma, St Louis, MO, USA). Aliquots of culture supernatant were collected and frozen at -80°C for subsequent virus titration and cytokine analysis. The supernatants were titrated on MDCK cells and the viral titre was quantitated as log_10_TCID_50_/ml. RNA was extracted from cells for analysis of cytokine gene expression. Ten hours after infection, replicate cell monolayers were fixed and analyzed by immuno-fluorescent staining specific for influenza virus nucleoprotein (DAKO Imagen, Dako Diagnostics Ltd, Ely, UK) to determine the proportion of cells infected.

### Quantification of cytokine mRNA by real-time quantitative RT-PCR

DNase-treated total RNA was isolated by means of RNeasy Mini kit (Qiagen, Hilden, Germany). The cDNA was synthesized from mRNA with poly(dT) primers and Superscript II reverse transcriptase (Life Technologies, Rockville, MD, USA) and quantified by real-time PCR analysis with a LightCycler (Roche, Mannheim, Germany). The mRNA for IP-10, interferon beta, IL-6, RANTES and tumor necrosis factor (TNF) alpha were quantitated using real-time RT-PCR. The oligonucleotide primers and methods used for real-time quantification of cytokines, viral matrix gene and the housekeeping gene product γ-actin mRNA have been described previously [12,24].

### Quantification of cytokine proteins by ELISA

The concentrations of IP-10, RANTES, interleukin 6 and interferon beta proteins in the primary human bronchial and alveolar epithelial cell supernatants were measured by a specific ELISA assay (R&D Systems, Minneapolis, MN, USA). Samples of culture supernatant were irradiated with ultraviolet light (CL-100 Ultra Violet Cross linker) for 15 min to inactivate any infectious virus before the ELISA assays were done. Previous experiments had confirmed that the dose of ultraviolet light used did not affect cytokine concentration as measured by ELISA (data not shown).

### Statistical analysis

The quantitative cytokine and chemokine mRNA and protein expression profile were compared using one-way ANOVA, followed by Bonferroni multiple-comparison test. Differences were considered significant at *p *< 0.05.

## Results

### *In vitro *infection of human type II pneumocytes

**Figure 1:**
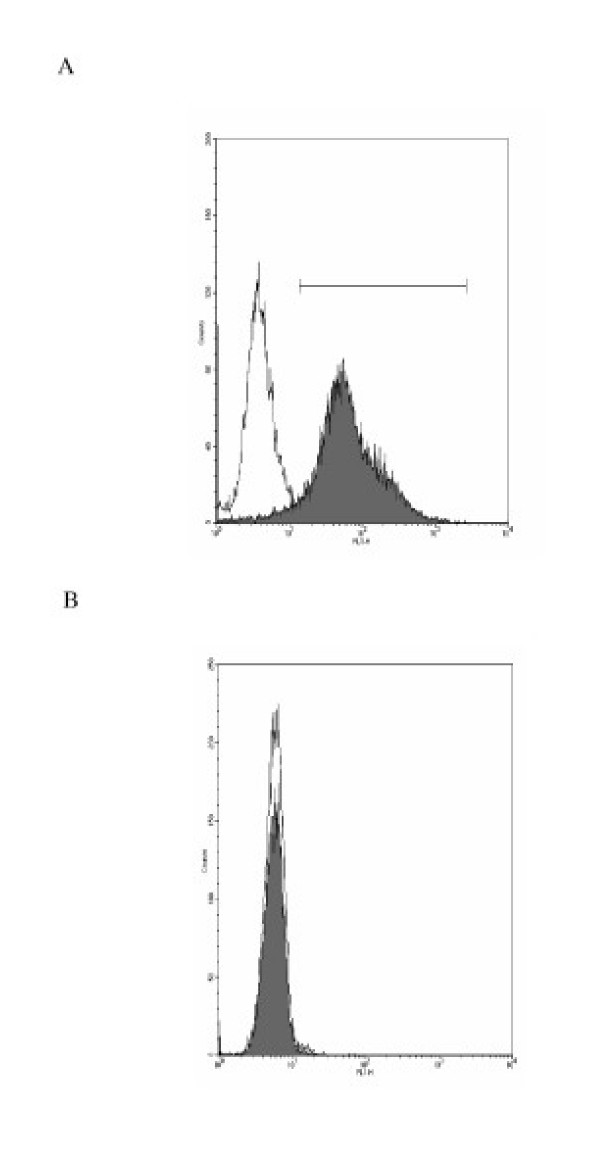
(A) Primary human type II pneumocytes were stained with antibody surfactant protein-C (shaded curve) and control antibody (unshaded curve) to confirm their identity. (B) Human type II pneumocytes isolated were stained with anti-CD14 FITC-conjugated antibodies (shaded curve) specific for macrophage surface antigen to check for any contaminant macrophage.

**Figure 2:**
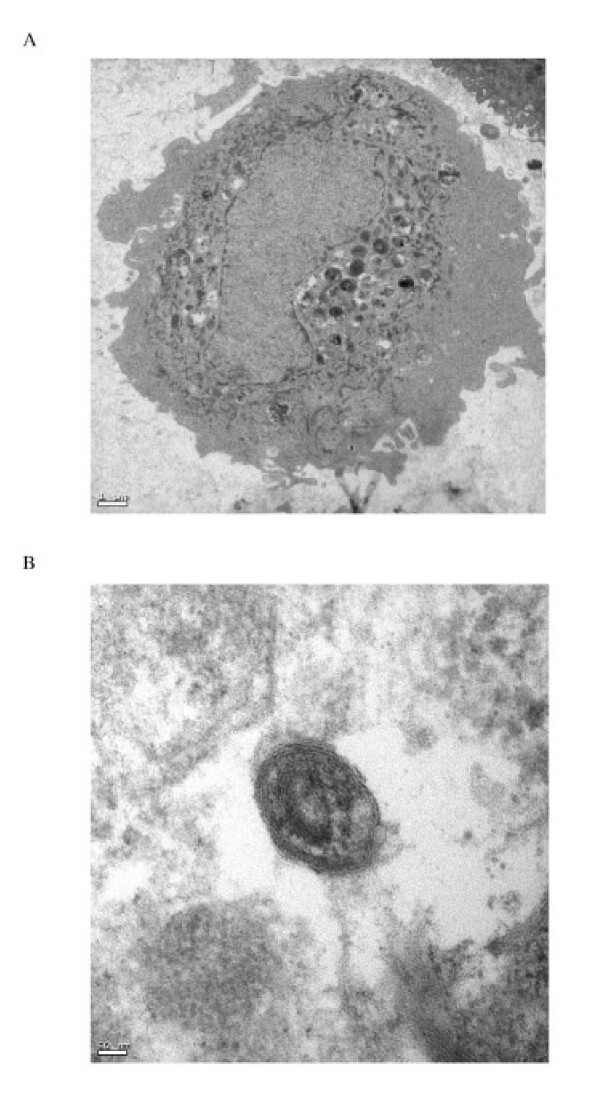
Transmission electron micrographs of human type II pneumocytes cultured *in vitro *(A) and the lamellar bodies in the cytoplasm demonstrated using higher magnification (B) (Bars: 1 μm and 50 nm respectively). The cells were scraped off the culture flask, fixed in 2% glutaraldehyde and embedded in Araldite resin.

Primary human type II pneumocyte yields were 3.5 ± 0.9 × 10^6 ^cells/g lung tissue at 92 ± 5% cell purity as demonstrated by the expression of the type II pneumocyte specific marker surfactant protein C (SP-C), lack of the monocyte/macrophage cell surface antigen (CD14) (Fig. 1A and 1B), and by staining for alkaline phosphatase activity. The contaminating cells were predominantly fibroblasts with monocyte/macrophage cells being less than 2%. Cell viability was 91 ± 7% (n = 13). Differences in age and sex of the lung donor had no apparent effects on cell isolation yields and the performance of the cells in culture. The isolated cells spread to form a confluent monolayer, exhibiting protruding nuclei surrounded by thin cytoplasmic extensions. The identity of the cells in culture as human type II pneumocytes was confirmed by demonstrating the presence of lamellar bodies and microvilli by thin section electron microscopy (Figure 2).

**Figure 4:**
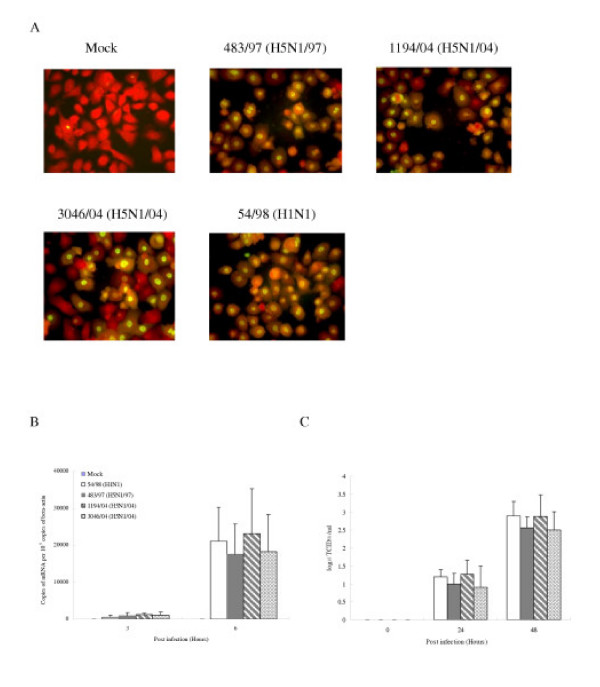
Infection of human type II pneumocytes with human influenza viruses. (A) Purified alveolar epithelial cells were fixed and analyzed by immunofluorescent staining specific for influenza virus nucleoprotein (×150). (B) The influenza M-gene mRNA profiles were assayed after infection. The concentrations of M-gene mRNA were normalized to those of β-actin mRNA in the corresponding sample. Means of duplicate assays are shown. (C) Alveolar epithelial cells were infected with human influenza viruses and the infectious virus yield (log_10_TCID_50_/ml) was determined in aliquots of supernatant collected at various times. Data are the means and the standard errors of independent experiments from three separate donors.

Previous studies have demonstrated that avian influenza viruses can infect human airway epithelial cells [25]. We first wanted to determine whether alveolar epithelial cells that constitutively reside in the lung can be infected with avian and human influenza viruses *in vitro*. The cells were infected with influenza A subtypes H5N1 (483/97, 1194/04 and 3046/04) and H1N1 (54/98) at a MOI of 2 and the proportion of cells expressing influenza A virus protein was analyzed at 10 hours post-infection by immunofluorescent staining using an antibody specific for the virus nucleoprotein (DAKO Imagen, Dako Diagnostics Ltd, Ely, UK). Similar proportions (93–100%) of type II pneumocytes infected with H5N1 and H1N1 virus had evidence of viral antigen (nucleoprotein) (Figure 4A). The quantification of influenza M-gene copies at 3 and 6 hours after infection in cells infected with H5N1 and H1N1 viruses showed comparable results at 3 and 6 hours post-infection (Figure 4B). Similarly, the infectious viral yield at 24 and 48 hours post-infection from alveolar epithelial cells infected with H5N1 and H1N1 viruses were not significantly different (Figure 4C).

### Induction of pro-inflammatory cytokines and chemokines in type II pneumocytes

We investigated the cytokine induction profile induced by H1N1 and H5N1 viruses in primary human type II pneumocytes. Specifically, we also wanted to determine if the two viruses differed qualitatively or quantitatively in the profile of cytokines induced. The mRNA of several cytokines and chemokines were quantified using quantitative RT-PCR at 3 hr and 6 hr post-infection (Table I). The mRNA levels of IP-10, interferon beta, RANTES and IL-6 were significantly up-regulated by influenza virus when compared with the mock infected cells, the genes for IP-10 and interferon beta being the most highly induced. There was no detectable TNF alpha induction in these epithelial cells (data not shown). Inactivation of the virus by ultraviolet irradiation prior to infection of the alveolar epithelial cells abolished cytokine induction (data not shown) suggesting that virus replication was required for cytokine induction.

**Table 1:** mRNA profile of cytokine and chemokine gene expression of primary culture of human type II pneumocytes 3 h and 6 h after infection with A/Hong Kong/483/97 (H5N1/97), A/Vietnam/1194/04, A/Vietnam/3046/04 (both H5N1/04) and A/Hong Kong 54/98 (H1N1) influenza viruses denoted as fold increase compared to mock infected cells.

Gene products	Ratio of expression over mock-infected cells							
	3 hours post infection				6 hours post infection			
	
	483/97 (H5N1/97) (-■-)^c^	1194/04 (H5N1/04) (-◆-)^c^	3046/04 (H5N1/04) (-×-)^c^	54/98 (H1N1) (-▲-)^c^	483/97 (H5N1/97) (-■-)^c^	1194/04 (H5N1/04) (-◆-)^c^	3046/04 (H5N1/04) (-×-)^c^	54/98 (H1N1) (-▲-)^c^

Interleukin 1	-1.2	0.3	0.9	1.1	1	0.9	0.8	-1.3
Interleukin 6	9.3*^a^	15.1*^b^	9.9*^a^	7.4*^a^	17.4*^b^	19.2*^b^	15.4*^b^	8.8*^a^
Interleukin 8	1	0.9	1.1	-1.2	-1.2	1.6	1.3	1.3
MCP-1	1.1	0.8	1.5	1	-1.2	1.7	1.3	1.3
TNF alpha	Not detectable				Not detectable			
RANTES	-1	9.5*^a^	2.2*	1.55	18.7*^b^	24.1*^b^	16.9*^b^	6.9*^a^
Interferon-alpha	1.1	0.8	0.6	1.2	-1.3	1.3	0.9	1.2
Interferon-beta	3.7*^a^	8.5*^a^	4.7*^a^	-0.8	22.1*^b^	26.3*^b^	18.7*^b^	13.3*^b^
IP-10	3.9*^a^	7.9*^a^	6.3*^a^	-3.5	37.9*^b^	46.8*^b^	29.7*^b^	8.1*^a^

Signals were normalized to the housekeeping gene, β-actin and expressed as a ratio over mock infected cells.

*Upregulation by two or more times over that of mock infection.

^a ^*p *< 0.01 and ^b ^*p *< 0.001 (Bonferroni multiple-comparison test).

^c ^Corresponding character symbols as shown in Figure 3 and 4.

**Figure 5:**
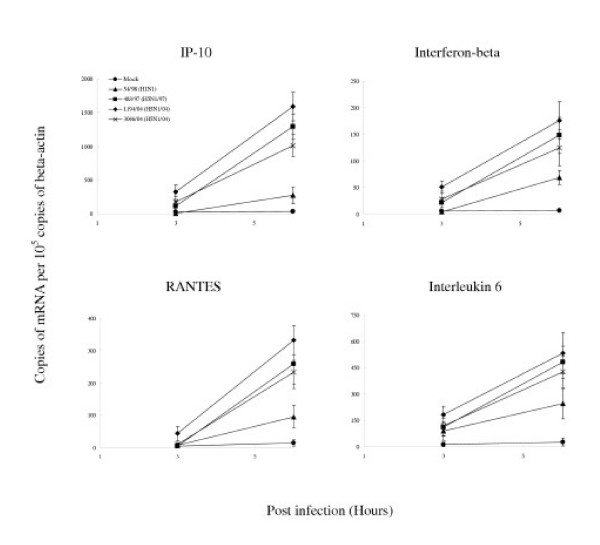
Cytokine and chemokine gene expression profile of influenza-virus-infected human type II pneumocytes by quantitative RT-PCR. Cytokine and chemokine mRNA concentration were assayed 3 h and 6 h after infection with A/Hong Kong/483/97 (H5N1/97), A/Vietnam/1194/04, A/Vietnam/3046/04 (both H5N1/04) and A/Hong Kong 54/98 (H1N1) influenza viruses or in mock infected cells. H5N1/97 and both H5N1/04 influenza viruses induced significantly higher levels of IP-10, interferon-beta, RANTES and IL-6 when compared to H1N1 infected cells at 6 hours post-infection (p < 0.001, Bonferroni multiple comparison test). The mRNA concentrations of cytokine and chemokine mRNA were normalized to those β-actin mRNA in the corresponding samples. Means and standard deviation from experiments from five different donors are shown

When compared with human H1N1 influenza virus, the H5N1/97 and H5N1/04 viruses differentially up-regulated the transcription of IP-10, interferon beta, RANTES and IL-6 to significantly higher levels (*p *< 0.001) (Figure 5). These differences were not explainable by a difference in proportion of cells infected as indicated by immunofluorescence for viral antigen or differences in virus titre (Figure 4). Furthermore, an increase in the multiplicity of infection of 54/98 (H1N1) virus from 2 to 10 did not result in cytokine mRNA concentrations similar to those induced by H5N1/97 and H5N1/04 (data not shown).

Broadly, there were two patterns of kinetics of cytokine gene transcription. Cytokines up-regulated from 3 hr post-infection onwards included IP-10, interferon beta and IL-6 whereas RANTES mRNA was only up-regulated at 6 hr post-infection (Table 1). The observations remained valid whether the cytokine mRNA expression data were analyzed with or without normalization for γ-actin mRNA concentrations.

### Infection and cytokine induction profile of primary human bronchial epithelial cells

**Figure 3:**
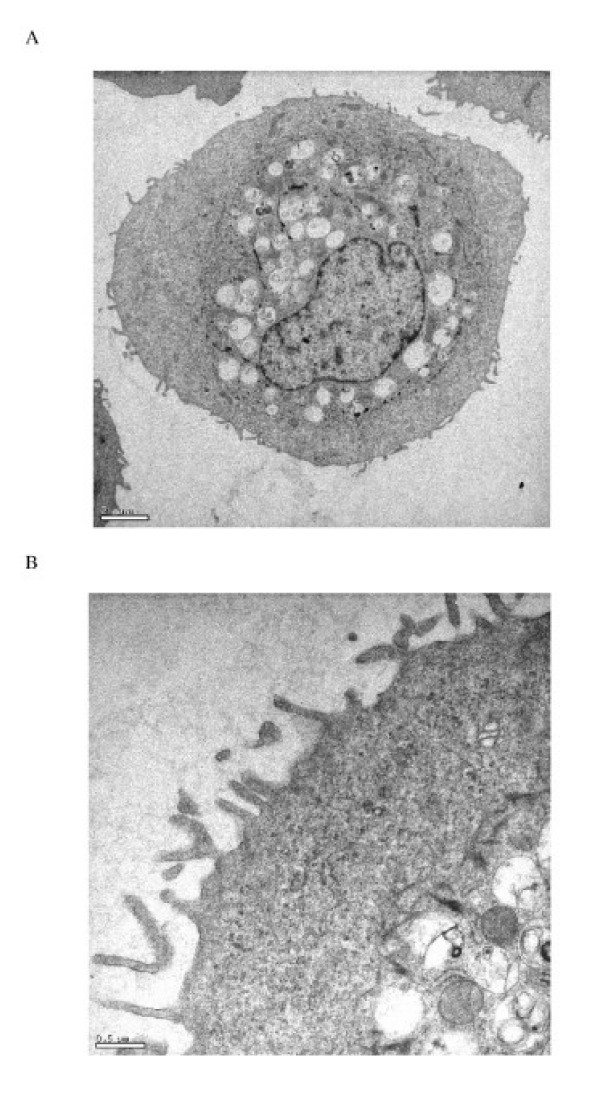
Transmission electron micrographs of human bronchial epithelial cells *in vitro *at low (A) and high (B) magnification (Bars: 2 μm and 0.5 μm respectively). The cells were scraped off the culture flask, fixed in 2% glutaraldehyde and embedded in Araldite resin.

**Figure 6:**
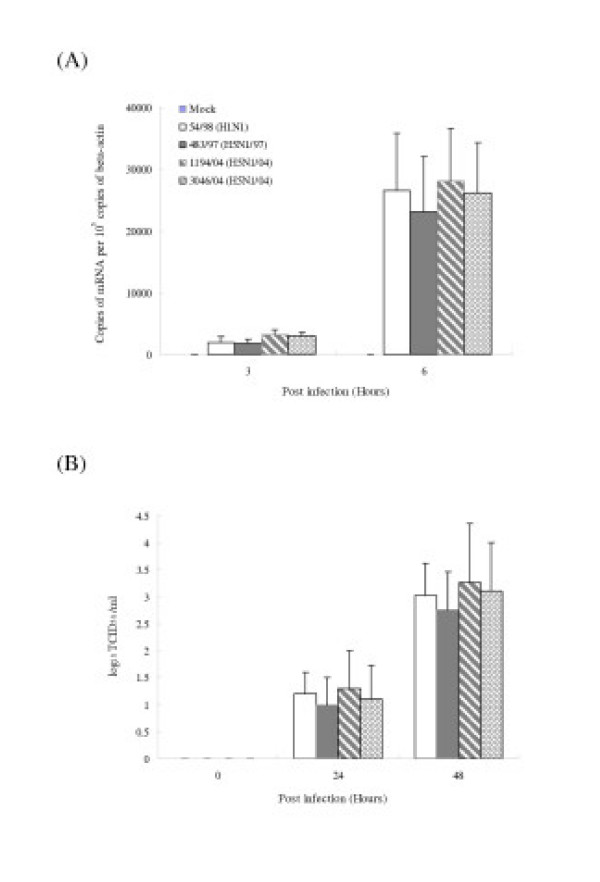
Infection of human bronchial epithelial cells with human influenza viruses. (A) The influenza M-gene mRNA profiles were assayed after infection. The concentrations of M-gene mRNA were normalized to those of β-actin mRNA in the corresponding sample. Means of duplicate assays are shown. (B) Virus yields (log_10_TCID_50_/ml) were determined in aliquots of supernatant collected from influenza-infected bronchial epithelial cells at various times. Data are the means and the standard errors of two independent experiments.

**Figure 7:**
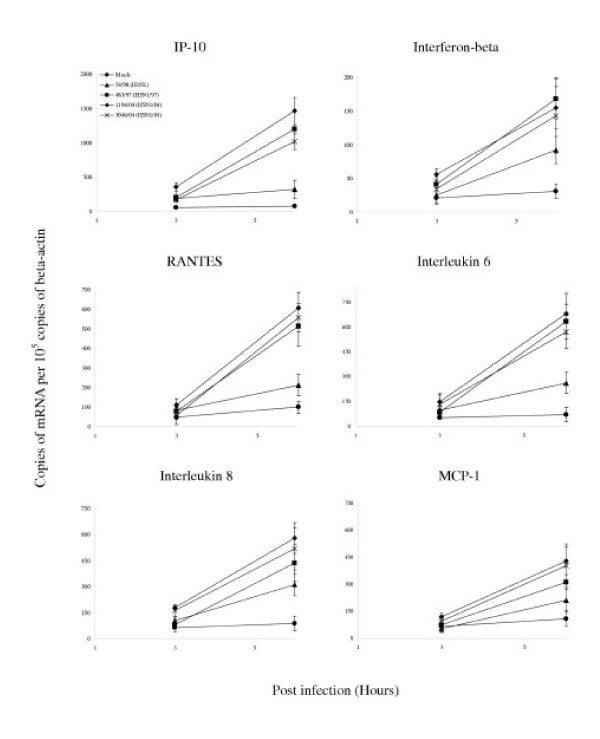
Cytokine and chemokine gene expression profile of influenza-virus-infected human bronchial epithelial cells by quantitative RT-PCR. Cytokine and chemokine mRNA concentration were assayed 3 h and 6 h after infection with A/Hong Kong/483/97 (H5N1/97), A/Vietnam/1194/04, A/Vietnam/3046/04 (both H5N1/04) and A/Hong Kong 54/98 (H1N1) influenza viruses or in mock infected cells. When compared with H1N1 infected cells, H5N1/97 and both H5N1/04 influenza viruses significantly up-regulated IP-10, RANTES and IL-6 (p < 0.001) and interferon beta (p < 0.01) at 6 hours post-infection (Bonferroni multiple comparison test). Both H5N1/04 viruses significantly up-regulated MCP-1 and IL-8 to levels higher than H1N1 and H5N1/97 infected cells (p < 0.05, Bonferroni multiple comparison test). The mRNA concentrations of cytokine and chemokine mRNA were normalized to those β-actin mRNA in the corresponding samples. Means and standard deviation of duplicate cultures and assays are shown.

The cytokine and chemokine profiles induced by H1N1, H5N1/97 and H5N1/04 viruses in primary human bronchial epithelial cells were similarly investigated. The identity of the cells in culture as human bronchial epithelial cells was confirmed by thin section electron microscopy (Figure 3). The overall gene expression profile was comparable to that seen with type II pneumocytes. The M-gene transcript copy numbers (Figure 6A) and infectious viral yields (Figure 6B) from bronchial epithelial cells infected with H5N1 and H1N1 viruses at an MOI of 2 were comparable. The H5N1/97 and H5N1/04 viruses differentially up-regulated the transcription of IP-10, interferon beta, RANTES and IL-6 to significantly higher levels than the human H1N1 virus (*p *< 0.001 for IP-10, RANTES and IL-6 and *p *< 0.01 for interferon beta) (Figure 7). In addition, the two H5N1/04 viruses (1194/04 and 3046/04) differentially up-regulated the transcription of monocyte chemotactic protein 1 (MCP-1) and IL-8 to significantly higher levels than the human H1N1 and H5N1/97 viruses (*p *< 0.05). None of the viruses induced TNF alpha in these cells.

### Secretion of cytokine proteins from bronchial and alveolar epithelial cells

**Figure 8:**
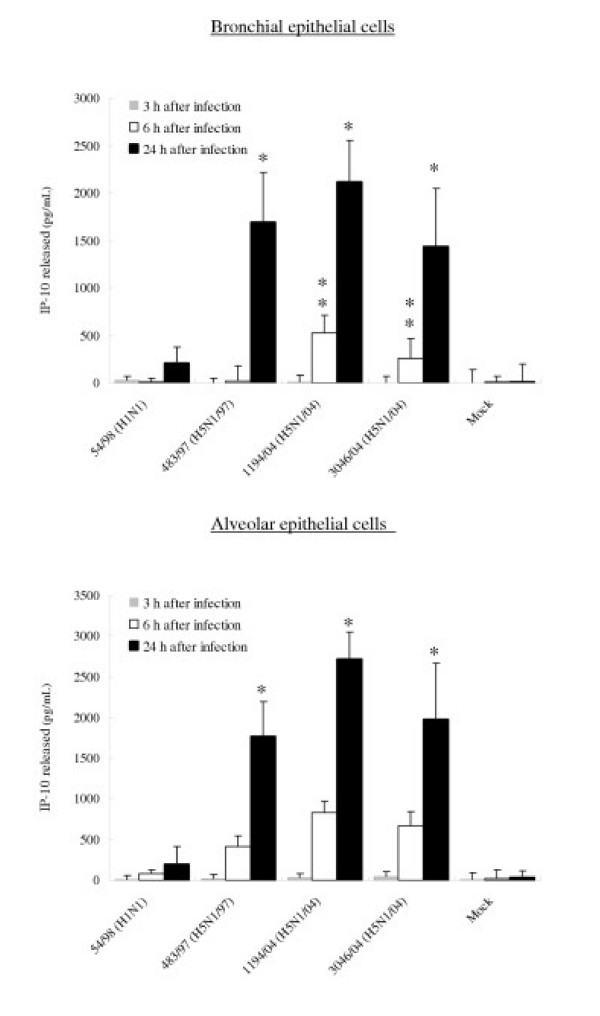
IP-10, Interleukin-6 and RANTES production by primary human bronchial and alveolar epithelial cells infected with A/Hong Kong/483/97 (H5N1/97), A/Vietnam/1194/04, A/Vietnam/3046/04 (both H5N1/04) and A/Hong Kong 54/98 (H1N1) influenza viruses or in mock infected cells. Culture supernatants from influenza virus-infected human respiratory epithelial cells collected at 3 h, 6 h and 24 h after infection with H5N1 and H1N1 viruses were tested by ELISA for IP-10 (Figure 8), Interleukin-6 (Figure 9) and RANTES (Figure 10). The IP-10, Interleukin-6 and RANTES mRNA levels were assayed at 3 h and 6 h post infection (data not shown) with results comparable with that shown in figure 5 and 7. The results from bronchial epithelial cells represent the means and standard deviations of three independent experiments (from the same donor). The means and standard deviations of the results from alveolar epithelial cells are based on experiments from six separate donors. * indicates p < 0.01 compared with mock and ** indicates p < 0.05 compared with H5N1/97 and H1N1 infected cells using the Bonferroni multiple comparison test.

**Figure 9:**
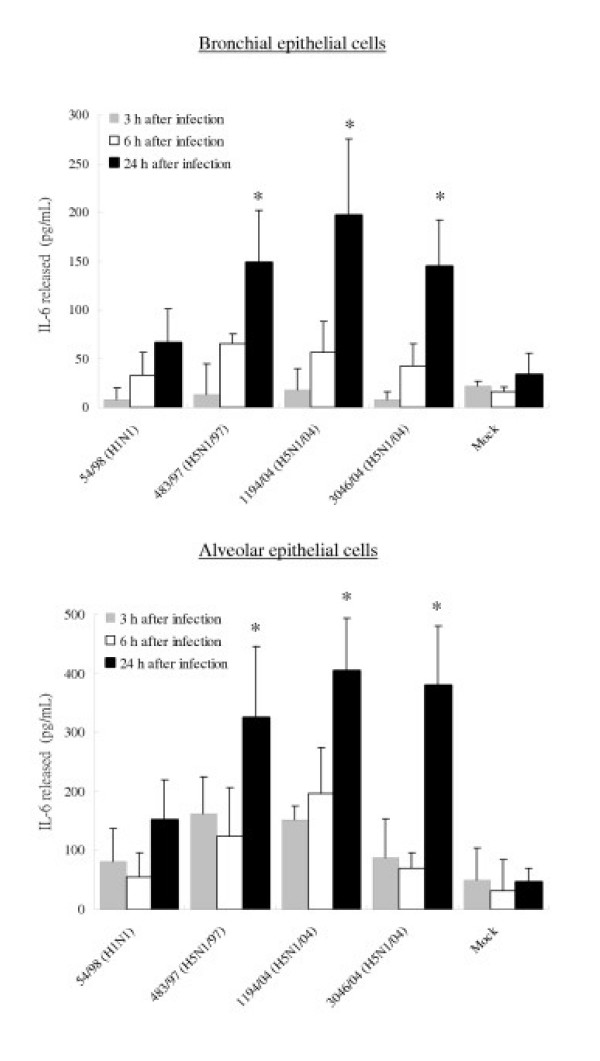
IP-10, Interleukin-6 and RANTES production by primary human bronchial and alveolar epithelial cells infected with A/Hong Kong/483/97 (H5N1/97), A/Vietnam/1194/04, A/Vietnam/3046/04 (both H5N1/04) and A/Hong Kong 54/98 (H1N1) influenza viruses or in mock infected cells. Culture supernatants from influenza virus-infected human respiratory epithelial cells collected at 3 h, 6 h and 24 h after infection with H5N1 and H1N1 viruses were tested by ELISA for IP-10 (Figure 8), Interleukin-6 (Figure 9) and RANTES (Figure 10). The IP-10, Interleukin-6 and RANTES mRNA levels were assayed at 3 h and 6 h post infection (data not shown) with results comparable with that shown in figure 5 and 7. The results from bronchial epithelial cells represent the means and standard deviations of three independent experiments (from the same donor). The means and standard deviations of the results from alveolar epithelial cells are based on experiments from six separate donors. * indicates p < 0.01 compared with mock and ** indicates p < 0.05 compared with H5N1/97 and H1N1 infected cells using the Bonferroni multiple comparison test.

**Figure 10:**
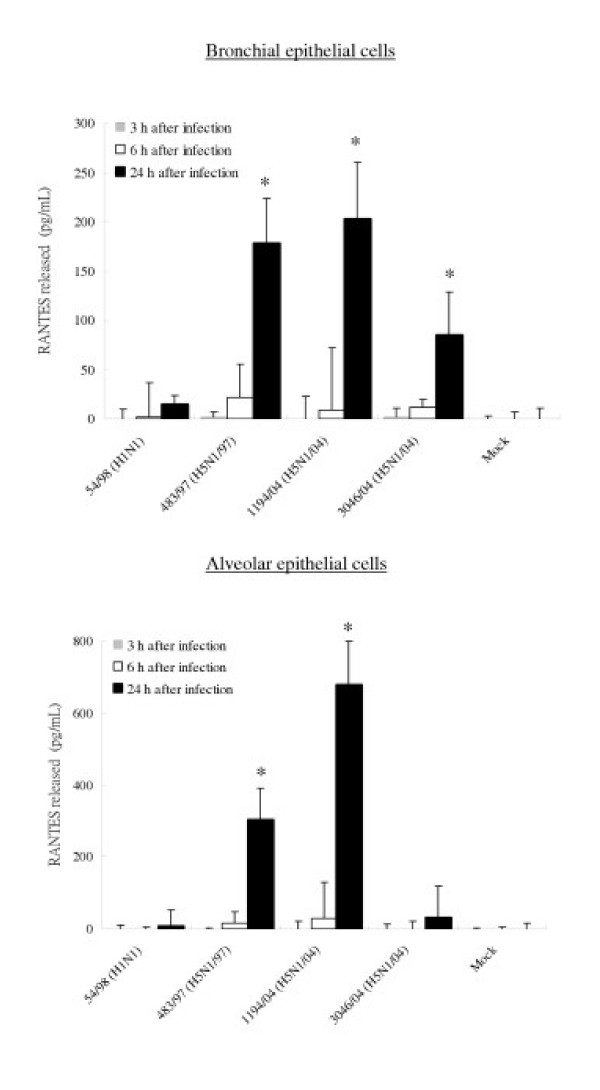
IP-10, Interleukin-6 and RANTES production by primary human bronchial and alveolar epithelial cells infected with A/Hong Kong/483/97 (H5N1/97), A/Vietnam/1194/04, A/Vietnam/3046/04 (both H5N1/04) and A/Hong Kong 54/98 (H1N1) influenza viruses or in mock infected cells. Culture supernatants from influenza virus-infected human respiratory epithelial cells collected at 3 h, 6 h and 24 h after infection with H5N1 and H1N1 viruses were tested by ELISA for IP-10 (Figure 8), Interleukin-6 (Figure 9) and RANTES (Figure 10). The IP-10, Interleukin-6 and RANTES mRNA levels were assayed at 3 h and 6 h post infection (data not shown) with results comparable with that shown in figure 5 and 7. The results from bronchial epithelial cells represent the means and standard deviations of three independent experiments (from the same donor). The means and standard deviations of the results from alveolar epithelial cells are based on experiments from six separate donors. * indicates p < 0.01 compared with mock and ** indicates p < 0.05 compared with H5N1/97 and H1N1 infected cells using the Bonferroni multiple comparison test.

To confirm that the observed differences of mRNA are reflected in levels of cytokine and chemokine secreted, the concentrations of the IP-10, RANTES, interleukin 6 and interferon-beta proteins were measured by ELISA in culture supernatants of infected bronchial and alveolar epithelial cells. The amount of IP-10 and IL-6 secreted by bronchial and alveolar epithelial cells infected with all three H5N1 viruses at 24 hours post infection were significantly higher (*p *< 0.01) than that secreted by cells infected with H1N1 virus (Figure 8 and 9). At 24 hours post infection, levels of IP-10 induced by H5N1/97 and both H5N1/04 viruses were comparable. However, at 6 hours post-infection, the recent H5N1/04 viruses 1194/04 and 3046/04 appeared to be even more potent at inducing IP-10 than H5N1/97 virus (*p *< 0.05) (Figure 8). RANTES protein secreted from bronchial and alveolar epithelial cells in response to H5N1/97 and 1194/04 (H5N1/04) were significantly higher than that induced by H1N1 virus. Although the level RANTES mRNA in 3046/04 (H5N1/04) infected cells at 6 hours post infection was significantly higher than those H1N1 infected cells, the RANTES protein secreted by these cells at 24 hours post infection was only increased 4 fold (*p *= 0.062; not significant) (Figure 5 and 10). We failed to detect any interferon-beta proteins secreted from the supernatants of bronchial and alveolar epithelial cells after influenza viruses infection (data not shown) but it should be noted that the limit of detection of the interferon-beta ELISA was high (250 pg/ml).

## Discussion

We found that the replication efficiency of the H5N1 and H1N1 viruses was similar in both primary human alveolar (Figure 4) and bronchial epithelial cells (Figure 6). Both influenza virus subtypes induced an IP-10, interferon beta, RANTES, and IL-6 responses. The cytokine induction was dependent on viral replication since UV-inactivated virus did not induce any effect. Interestingly, we found that H5N1/97 and 1194/04 (H5N1/04) viruses were more potent inducers of IP-10, interferon-beta, RANTES and IL-6 mRNA and protein than the human H1N1 virus (Figure 5, 7, 8 to 10). Thus, the observed differences of mRNA are reflected in levels of cytokine and chemokine proteins secreted (Figure 8 to 10). The results with 3046/04 (H5N1/04) were generally similar to 1194/04 (H5N1/04) with the exception that the levels of RANTES protein in type II pneumocytes was not significantly elevated when compared with H1N1 virus infected cells (Figure 10) although the mRNA levels were (Figure 5). Our inability to detect any interferon-beta proteins in our experiments in spite of marked induction of mRNA is probably related to the limited sensitivity of the interferon beta ELISA. A more sensitive bioassay for interferon-beta may be required for this purpose. The type II pneumocytes used in these experiments were derived from a total of 13 donors and each set of experimental data is based on the results of at least three separate experiments from three donors therefore excluding a donor specific artifact. The bronchial epithelial cells were purchased from a commercial source and comes from one donor. However, since the results from these cells are broadly in line with those from the type II pneumocytes, again, we think that donor specific artifacts are unlikely to explain the results we have obtained. Finally, these results are also comparable to our previous observations from primary human monocyte derived macrophages [12] with the exception that in contrast to macrophages, no TNF alpha and IL-1 beta was induced in respiratory epithelial cells by any of the viruses tested.

This differential hyper-induction of cytokines was not explained by differences in the replication kinetics between the two virus subtypes. H5N1 viruses isolated from patients with H5N1 disease in Hong Kong in 1997, Vietnam in 2004 and human influenza viruses of the H1N1 subtype all replicate with similar efficiency. Increase in the MOI of the H1N1 virus did not result in an increase of cytokine responses to levels comparable to that of the H5N1 viruses. The cellular mechanisms underlying this differential cytokine hyper-induction by H5N1 viruses are presently poorly understood. Studies on the transformed bronchial epithelial cell line A549 previously demonstrated that toll-like receptor 3 (TLR-3) is involved in the influenza virus A initiated cytokine responses [27]. It remains to be determined whether H5N1 viruses also act via TLR-3 signaling in primary human epithelial cells.

Cytokine and chemokine responses *in vivo *result from autocrine and paracrine interactions involving many cell types. Chemokines such as IP-10 and MCP-1 are macrophage chemo-attractants and mediate the inflammatory response by further recruitment of circulating leukocytes into the inflamed tissue. We have previously demonstrated that IP-10 and MCP-1 are up-regulated in primary human macrophage by SARS-CoV [28]. The strong induction of chemokines in the lung micro-environment might explain the prominent macrophage infiltrate observed in the lungs of patients with fatal H5N1 [4] as well as SARS [29].

RANTES attracts monocytes, eosinophils, basophils and T cells, and selectively CD4+ T cells. Its production from the bronchial epithelial cells contributes to the infiltration of the inflammatory cells in airway viral infection [18]. IL-6 is a multifunctional cytokine that can regulate immune and inflammatory responses involved in the activation, growth and differentiation of T-cells [30] and can contribute to T cell mediated inflammatory reactions. In fact, autopsy examination showed an increased CD3+ T cells in the interstitium of the lung from patients with H5N1 diseases [4]. In addition, IL-6 has been shown to be released by macrophages and epithelial cells during lung injury [31] and the effects of IL-6 are synergistic with those of IL-1 and TNF-alpha [32]. We have previously demonstrated that other proinflammatory cytokines such as IL-1, TNF-alpha and IL-6 are hyper-induced in H5N1 infected macrophages [12]. Therefore, the differential up-regulation of IL-6 expression in human respiratory epithelial cells and the cytokines induced in macrophages by H5N1 viruses may contribute synergistically to the pathogenesis of human H5N1 disease.

The H5N1 viruses have continued to reassort, acquiring different internal genes from other influenza viruses of avian origin [33,34]. The H5N1/04 viruses, A/Vietnam/1194/04 and A/Vietnam/3046/04 represent the Z genotype viruses that emerged as the dominant virus genotype affecting poultry in south-east Asia [27,35]. Thus there appears to be an association between the property of hyper-inducing cytokines and high virulence. Additionally, in pig epithelial cells, H5N1/97 viruses were found to resist the antiviral effects of interferon [36] and this may also be relevant in pathogenesis. It is notable that patients with avian influenza (H5N1) disease appeared to have higher levels of IP-10 in their sera than those with infections with the human influenza viruses [4] providing *in vivo *data that parallels our present findings *in vitro*. Studies on recombinant viruses bearing the HA and NA of the 1918 "Spanish flu" pandemic virus showed that these viruses have enhanced virulence for mice and induce higher levels of macrophage-derived chemokines *in vivo *in mice [37]. However, such observations of hyper-induction of cytokines *in vivo *may simply reflect more extensive replication of the respective virus. The studies *in vitro *with H5N1 viruses exclude such potential confounding factors and it would be relevant to study the cytokine profiles of the 1918 recombinant viruses in *in vitro *models similar to those described here.

## Conclusion

H5N1 subtype influenza A viruses associated with human disease are more potent than human H1N1 virus at inducing proinflammatory cytokines and chemokines, including IP-10, interferon beta, IL-6 and RANTES, from human primary alveolar and bronchial epithelial cells infected *in vitro*. Previous findings showed that H5N1/97 viruses also hyper-induce cytokines from macrophages and that patients with H5N1 disease have high levels of IP-10 and other chemokines in the serum. These findings may be relevant to the pathogenesis of H5N1 disease. The recent re-emergence of H5N1 disease in humans is a cause for renewed pandemic concern and highlights the need for a better understanding of the pathogenesis of human H5N1 disease. Such understanding will lead to new strategies for managing human H5N1 disease and enhance our preparedness to confront pandemic influenza, whether from H5N1 or other influenza A subtypes.

## Competing interests

The author(s) declare that they have no competing interests.

## Authors' contributions

JSM Peiris, MCW Chan and CY Cheung conceived the study, planned the overall experimental design and wrote the manuscript. MCW Chan carried out the experiments; MCW Chan, CY Cheung and YO Chan carried out experiments in the BL3 laboratory and RWY Chan assisted in experiments in the BL2 laboratory. WH Chui provided the lung biopsy specimens, SW Tsao helped to develop the methods for primary culture of the human alveolar epithelial cells, JM Nicholls advised on morphogical studies, and LLM Poon and Y Guan advised in experimental design. All authors critically reviewed the manuscript.
